# Cancer-associated fibroblasts and their role in tumor progression

**DOI:** 10.18699/VJGB-22-03

**Published:** 2022-02

**Authors:** M.S. Ermakov, A.A. Nushtaeva, V.A. Richter, O.A. Koval

**Affiliations:** Institute of Chemical Biology and Fundamental Medicine of the Siberian Branch of the Russian Academy of Sciences, Novosibirsk, Russia; Institute of Chemical Biology and Fundamental Medicine of the Siberian Branch of the Russian Academy of Sciences, Novosibirsk, Russia; Institute of Chemical Biology and Fundamental Medicine of the Siberian Branch of the Russian Academy of Sciences, Novosibirsk, Russia; Institute of Chemical Biology and Fundamental Medicine of the Siberian Branch of the Russian Academy of Sciences, Novosibirsk, Russia; Novosibirsk State University, Novosibirsk, Russia

**Keywords:** cancer-associated fibroblasts, epithelial-to-mesenchymal transition, carcinoma, hypoxia, опухоль-ассоциированые фибробласты, эпителиально-мезенхимальный переход, карцинома, гипоксия

## Abstract

The stromal elements of a malignant tumor can promote cancer progression and metastasis. The structure of the tumor stroma includes connective tissue elements, blood vessels, nerves, and extracellular matrix (ECM). Some of the cellular elements of the tumor stroma are cancer-associated f ibroblasts (CAFs). The origin and function of CAFs have been actively studied over the past thirty years. CAFs produce collagen, the main scaffold protein of the extracellular matrix. Collagen in the tumor stroma stimulates f ibrosis, enhances the rigidity of tumor tissue, and disrupts the transmission of proliferation and differentiation signaling pathways. CAFs control tumor angiogenesis, cell motility, tumor immunogenic properties, and the development of resistance to chemo- and immunotherapy. As a result of metabolic adaptation of rapidly growing tumor tissue to the nutrients and oxygen deprivation, the main type of energy production in cells changes from oxidative phosphorylation to anaerobic glycolysis. These changes lead to sequential molecular alterations, including the induction of specif ied transcriptional factors that result in the CAFs activation. The molecular phenotype of activated CAFs is similar to f ibroblasts activated during inf lammation. In activated CAFs, alpha-smooth muscle actin (α-SMA) is synthetized de novo and various proteases and f ibronectin are produced. Since CAFs are found in all types of carcinomas, these cells are potential targets for the development of new approaches for anticancer therapy. Some CAFs originate from resident f ibroblasts of the organs invaded by the tumor, while others originate from epithelial tumor cells, which are undergoing an epithelial-mesenchymal transition (EMT). To date, many molecular and metabolic inducers of the EMT have been discovered including the transforming growth factor-beta (TGF-β), hypoxia, and inf lammation. This review classif ies modern concepts of molecular markers of CAFs, their functional features, and discusses the stages of epithelial- mesenchymal transition, and the potential of CAFs as a target for antitumor therapy

## Abstract (ru)

Стромальные элементы опухоли могут стимулировать прогрессию опухолевого роста и метастазирование. В структуру опухолевой стромы входят соединительнотканные элементы, сосуды, нервы и внеклеточный матрикс. Одним из клеточных элементов стромы опухоли являются опухоль-ассоциированные фибробласты (ОАФ), происхождение и функции которых активно изучают на протяжении последних тридцати лет. ОАФ продуцируют основной каркасный белок внеклеточного матрикса – коллаген, избыток которого в строме опухоли стимулирует фиброз, повышает жесткость опухолевой ткани и нарушает передачу сигналов пролиферации и дифференцировки. ОАФ контролируют ангиогенез в опухоли, подвижность опухолевых клеток, иммуногенные свойства опухоли и развитие резистентности к химиопрепаратам и иммунотерапии. В результате метаболической адаптации быстрорастущей опухолевой ткани к нехватке питательных веществ и кислорода, при конверсии основного типа производства энергии в клетках с окислительного фосфорилирования на анаэробный гликолиз инициируются молекулярные изменения, в том числе индукция определенных транскрипционных факторов, которые обусловливают активацию ОАФ. Молекулярный фенотип активированных ОАФ имеет сходство с фибробластами, активированными в процессе воспаления. В активированных ОАФ происходит de novo синтез альфа-актина гладкой мускулатуры (α-SMA), продукция различных протеаз и фибронектина. Поскольку ОАФ обнаружены во всех типах карцином, эти клетки могут быть мишенями для разработки новых подходов противоопухолевой терапии. Часть ОАФ происходит из резидентных фибробластов пораженного опухолью органа, другие берут свое начало из опухолевых эпителиоцитов, претерпевая эпителиально-мезенхимальный переход. На сегодняшний день обнаружено большое количество молекулярных и метаболических индукторов эпителиально-мезенхимального перехода, к которым относят трансформирующий фактор роста бета (TGF-β), гипоксию и воспаление. В данном обзоре систематизированы современные представления о молекулярных маркерах ОАФ, их функциональных особенностях, этапах эпителиально-мезенхимального перехода и обсуждается возможность использования ОАФ в качестве мишеней для противоопухолевой терапии.

## The biology of cancer-associated f ibroblasts

Modern concept of tumor morphology postulates that solid tumors are formed by epithelial and stromal cells, such as fibroblasts, endothelial cells, and immune cells (Wang et al., 2017). Stromal cells with a fibroblast-like phenotype, the so-called cancer-associated fibroblasts (CAFs), in contrast to normal fibroblasts, contain various chromosomal abnormalities, such as duplications, multiple rearrangements, and even the loss of entire chromosomes (Hosein et al., 2010). CAFs control tumor angiogenesis, motility and metastasis of cancer cells, tumor immunogenic properties, and the development of resistance to chemotherapy and immunotherapy (Tripathi et al., 2012; Alkasalias et al., 2018; Nushtaeva et al., 2018).

A meta-analysis of the clinical relevance of the tumor stroma has demonstrated the association of high CAFs content with the advanced stages of tumor progression, as well as with the high risk of local recurrence after tumor resection (Knops et al., 2020).

## Heterogeneity of CAFs’ percussors cells

In 1995, a heterogeneous origin of CAFs was hypothesized by Rønnov-Jessen and colleagues, who showed that breast cancer CAFs can originate from resident fibroblasts, vascular smooth muscle cells, and pericytes (Rønnov-Jessen et al., 1995). To date, it has been shown that precursors of mesenchymal cells from the red bone marrow, endothelial and epithelial cells, resident fibroblasts of the affected tissue, adipocytes and vascular adventitia cells can be sources of CAFs (Puré, Hingorani, 2018; Yin et al., 2019). For the initiation of the CAFs phenotype in some progenitor cells, additional stimulation with cytokines and growth factors, such as transforming growth factor beta (TGF-β), fibroblast growth factor (FGF), and other signaling molecules is required (Table 1) (Bordignon et al., 2019).

**Table 1.**
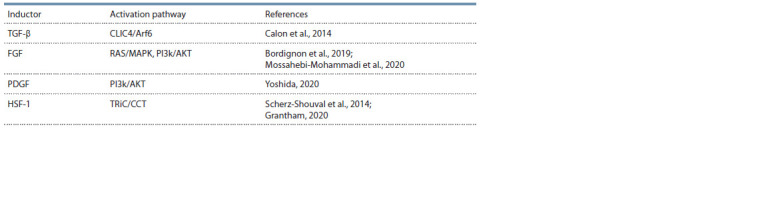
The inducers of CAFs phenotype Notе. PDGF – platelet derived growth factor; HSF-1 – heat shock factor 1; CLIC4/Arf6 – intracellular chloride channel 4, ADP-ribosylation factor 6; RAS/MAPK – mitogen activated protein kinase; PI3k/AKT – phosphoinositide 3-kinase, alpha serine/threonine protein kinase; TRiC/CCT is a chaperonin containing TCP-1.

**Fig. 1.**
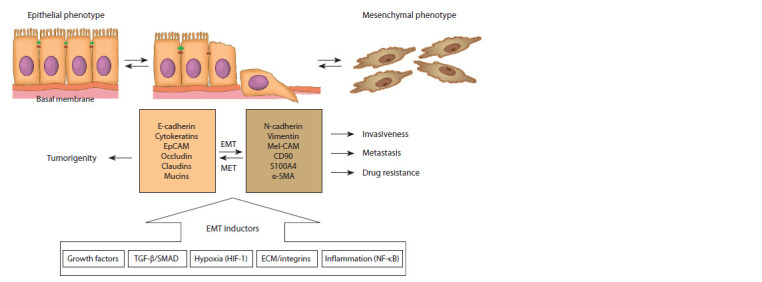
Cellular and molecular changes during EMF and MET. EpCAM – epithelial adhesion molecule; Mel-CAM – melanoma adhesion molecule; α-SMA – alpha smooth muscle actin; HIF-1 – hypoxia inducible factor 1.

Tumor epithelial cells can undergo transformation into CAFs via the epithelial-mesenchymal transition (EMT) (Fig. 1). EMT is a dynamic process of transdifferentiation of epithelial cells into fibroblast-like cells. The EMT plays an important role not only in cancer, but also in embryogenesis and regeneration. In particular, EMT occurs in embryonic stem cells producing mesoderm and neural crest, and in skin cells during wound healing (Kim et al., 2017). Dynamic changes in cell morphology during EMT are caused by changes in the regulatory genes’ expression with production of certain proteins. These proteins are considered as EMT markers (see Fig. 1). Among these markers, the most significant are N-cadherin and vimentin, which are responsible for the rearrangement of the cytoskeleton and the change in the shape of the cell, as well as the change in cell-to-cell and cell-toextracellular matrix (ECM) interactions (Massagué, 2008;Ye, Weinberg, 2015).

In addition to molecular inducers of EMT, the important role of hypoxic conditions has been shown. Hypoxia activates EMT via the binding of the hypoxia-inducible factor (HIF-1) to the promoters of genes responsible for EMT activation. HIF-1 has been shown to increase the expression of the transcription factors genes of the zinc finger motif family such as ZEB1, Snail and SLUG. Overexpression of these factors is associated with the mesenchymal phenotype and a decrease in the abundance of epithelial cell markers – E-cadherin and type 1 tight junction protein (TJP1 or ZO-1) (Nushtaeva et al., 2019; Tam et al., 2020).

Endothelial cells of tumor vessels can undergo an endothelial- mesenchymal transition (EndMT) and acquire the phenotype and functional features of CAFs with the loss of endothelial cells molecular markers, such as the endothelial cell/platelet adhesion molecule (CD31), and the acquisition of markers specific for mesenchymal cells, such as α-SMA and fibroblast specific protein 1 (FSP-1) (Zeisberg et al., 2007).

An important component of breast cancer stroma are adipose cells, which can transform into tumor-associated adipocytes, and then into CAFs. Such changes are accompanied by an increase in the expression of molecular markers of mesenchymal cells, including, PPARG (receptors induced by peroxisome activators gamma), RUNX-2 (transcription factor containing the Runt type 2 DNA-binding domain), and SOX9 (transcription factor of the HMG family DNA-binding proteins) (Bochet et al., 2013; Liu et al., 2021).

Using the model of prostate cancer, it was shown that mesenchymal stem cells (MSCs) can differentiate into CAFs after the activation of the chemokine receptor type 6 (CXCR6) by its ligand CXCL16. Moreover, the activation of CXCR6 results in the secretion of stromal factor-1 (CXCL12) involved in EMT (Jung et al., 2013). Weber and colleagues also showed that the extracellular structural protein osteopontin (OPN), which plays a key role in bone formation, activates TGF-β gene expression in integrin-dependent MSCs to maintain the phenotype of CAFs in breast cancer. Interestingly, even specialized cells such as Ito cells in the liver, pancreatic stellate cells, and mammary myofibroblasts can acquire the phenotype of CAFs (Weber et al., 2015). These examples illustrate a wide range of cells that, responding to the molecular changes in a tumor, are able to acquire the CAFs’ phenotype and, as a consequence, be involved in tumor homeostasis.

## Markers of cancer-associated fibroblasts

The involvement of CAFs in carcinogenesis and tumor progression makes them a potential target for the development of novel therapeutic approaches. A potentially clinically significant marker of CAF is the transmembrane mucin-like protein podoplanin (PDPN) (Table 2); to date, PDPN has been described as a marker of lymphoid capillary progenitor cells and CAFs in lung cancers. Expression of podoplanin was showed in 54 (30.5 %) out of 177 CAFs’ populations studied in the work of Yurugi et al. Interestingly, all podoplanin-positive CAFs correlated with invasiveness of adenocarcinomas, while a podoplanin-negative phenotype was shown only in noninvasive adenocarcinomas (Yurugi et al., 2017).

**Table 2.**
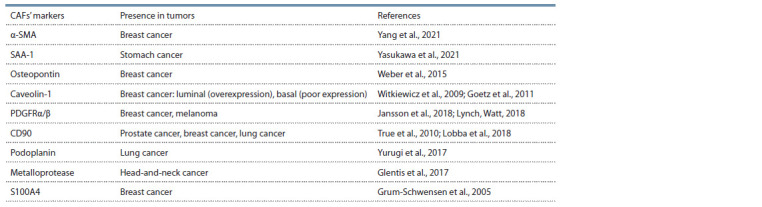
Potential molecular markers of CAFs Notе. α-SMA – alpha-smooth muscle actin; PDGFRα/β – platelet-derived growth factor receptors α/β.

Platelet-derived growth factor receptors α/β are important markers of CAFs. PDGFRα/β belong to the 3rd class of tyrosine kinases and are activated by interaction with the PDGF ligand. PDGFR regulates the organogenesis of various systems during embryogenesis; however, the significance of the PDGFRα and -β receptors activation in tumors is still poorly understood. It has been shown that the expression of the PDGFRβ receptor is increased in the tumor microenvironment cells, where platelet growth factor activates CAFs and, probably, stimulates cancer progression (Anderberg et al., 2009). PDGFRα-positive CAFs have been found in the stroma of melanoma, suggesting that these CAFs originate from resident fibroblasts as a result of their activation (Lynch, Watt, 2018). Serum amyloid A (SAA-1) protein is one of the potential targets of CAFs; its expression and involvement in tumor progression has been shown in CAFs from gastric tumors (Yasukawa et al., 2021).

In the search for specific markers of the tumor stroma cells, among the CAFs of prostate adenocarcinoma, an increased content of the surface protein with a single V-domain of immunoglobulin (CD90), initially found on T cells and neurons, was identified as a specific marker. The high level of CD90 on the cell surface differentiates the tumor-associated stroma and “benign” stroma. Since CD90 expression was shown only in tumor associated fibroblasts, this marker is a potential target for therapy (True et al., 2010).

Certain CAFs proteins can be prognostic markers of tumor invasiveness. One of these markers is a protein from the family of low molecular weight calcium-binding proteins of the S100 – S100A4 family (Fei et al., 2017). S100 family proteins have both intracellular and extracellular activity due to maintaining the calcium balance and Ca2+-dependent processes. S100A4 activates a cascade of reactions associated mainly with the secretion of pro-inflammatory cytokines and the expression of growth factors, extracellular matrix proteins, metalloproteinases, and others. Intracellular activity of S100A4 is of particular interest, and is associated with the enhancement of the invasive capabilities of tumor cells, their escape from apoptosis, and the stem phenotype of the cells (Ambartsumian et al., 2019). During the study of the role of S100A4 in tumor progression, it was shown that suppression of S100A4 decreased tumor growth (Joyce, Pollard, 2009; Grum-Schwensen et al., 2015). The role of stromal cells that secrete S100A4 was shown in the MMTV-PyVmT mouse model with the S100A4 knocked-out gene during orthotopic co-transplantation of CSML100 mouse mammary adenocarcinoma cells and MEF mouse embryonic fibroblasts. MEF cell lines were obtained by spontaneous immortalization of primary embryonic fibroblasts from mouse embryos with the S100A4+ and S100A4– phenotypes. Upon co-transplantation of tumor cells and fibroblasts with the S100A4– phenotype in syngeneic mice, no metastases were formed, however, upon transplantation of S100A4+ fibroblasts, the metastatic potential of tumor cells returned. S100A4+ fibroblasts were characterized by increased mobility and invasiveness compared to S100A4– fibroblasts, as well as the ability to secrete S100A4 into the tumor microenvironment (Grum-Schwensen et al., 2005).

To date, it is clear that the expression of certain CAFs markers does not unambiguously predict the aggressiveness of the tumor. For example, the loss of caveolin-1 in breast cancer CAFs has been shown to be associated with a poor prognosis because the population of these cells stimulates the growth of triple negative (ER-/PR-/HER2-) breast cancer cells (Witkiewicz et al., 2009). In a parallel study, the expression of caveolin-1 in the breast cancer CAFs stimulated the remodeling of the tumor microenvironment, thereby facilitating the invasion of malignant cells and an increased invasiveness level correlated with the metastatic potential of the tumor (Witkiewicz et al., 2009; Goetz et al., 2011). These contradicting results indicate the diverse role of caveolin-1 in histologically different tumors. More studies should be made to determine caveolin-1 as a tumor prognostic marker.

## Role of cancer-associated f ibroblasts in tumor progression

CAFs-dependent stimulation of the tumor cells proliferation and their invasion is of particular interest in the study of tumor. This interest is primarily due to the fact that even in the precancerous phenotype of epithelial cells, some resident fibroblasts are already transformed into CAFs (Liotta, Kohn, 2001). Fibroblasts from intestinal tumors and polyps were a good model to confirm the contribution of stromal cells to tumor growth and progression. These fibroblasts were shown to stimulate the proliferation of tumor and polyp cells (Mukaida, Sasaki, 2016).

The interaction of tumor epithelial cells with CAFs was analyzed by comparing the histological picture of various types of gastric cancer. In a study by Orimo and Weinberg, it was demonstrated that in the case of diffuse gastric cancer, CAFs and epithelial cells are more closely spaced, while in the intestinal type, CAFs form a stroma-like matrix, due to which tumor epithelial cells retain their glandular structure (Orimo, Weinberg, 2006).

Using the model of heterogeneous 3D spheroids, consisting of breast cancer epithelial cells and fibroblasts, Dang and colleagues showed that CAFs stimulated the migration of tumor cells of basal breast cancer (ER-/PR-/HER2-). Interestingly, this effect was not observed in the models of luminal breast cancer types (ER+/PR+/HER2+, ER+/PR+/HER2-) (Dang et al., 2011). These data are consistent with clinical observations indicating a higher percentage of metastasis in triplenegative breast cancer compared with other types of breast tumors. However, which factors make cells of basal breast cancer sensitive to CAFs stimulation remain undiscovered (Al-Mahmood et al., 2018).

In order to reach the blood and lymphatic streams, tumor cells should pass through the basement membrane (BM), separating them from connective tissue and vessels. Thus, CAFs are able to synthesize metalloproteinases – endopeptidases capable of destroying proteins of all types of the BM extracellular matrix (Gonzalez-Avila et al., 2019). In 2017, Glentis and colleagues revealed a metalloproteinase-independent CAFssupported overcome of BM by tumor cells. They demonstrated the ability of CAFs to stretch BM with the formation of pores, and through these pores, epithelial tumor cells and CAFs can migrate into the bloodstream and form metastases in distant organs. Interestingly, BM regions with low expression of laminin and type IV collagen exhibited the highest tendency for stretching (Glentis et al., 2017). This alternative CAFsdependent migration pathway explains the ineffectiveness of the metalloproteinase inhibitors application in patients with head and neck tumors.

The paracrine secretion of IL-1α by CAFs in bladder cancer with further activation of the Wnt pathway in tumor cells is the perfect illustration of the pro-carcinogenic role of CAFs (Yang et al., 2021). Moreover, bladder cancer СAFs secreting IL-8 are able to stimulate the secretion of neuropilin-1, which enhances the proliferation of tumor cells and is one of the potential prognostic markers of malignancy (Chen C. et al., 2020). Interestingly, recent studies have shown that neuropilin-1 may be a co-factor in the induction of EMT (Chen Z. et al., 2020).

**Fig. 2.**
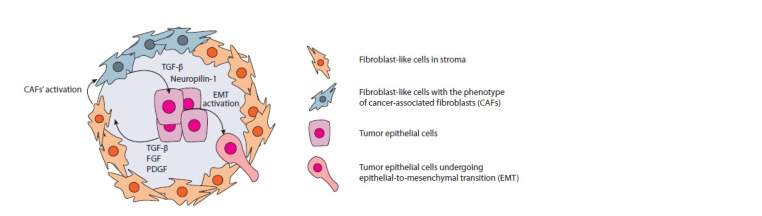
The interactions of cancer epithelial cells and stromal elements in the tumor TGF-β – transforming growth factor-β; FGF – fibroblast growth factor; PDGF – platelet growth factor.

Paracrine stimulation of the epithelial-mesenchymal transition in tumor epithelial cells by CAFs is an important factor contributing to tumor progression. The central mechanism of EMT in the tumor is the TGF-β/Smad pathway activation, induced by TGF-β from stromal fibroblasts (Fig. 2) (Yu et al., 2014). The Smad factor is a transcription factor that controls the expression of EMT genes. Vered and colleagues showed that cells with EMT markers are found in primary foci of squamous cell carcinoma of the tongue as well as in regional lymph nodes metastases. This confirms the importance of CAFs in the induction of metastasis and in the formation of a secondary tumor node (Vered et al., 2010).

The central mechanism of the EMT activation in ovarian cancer is the induction of the CXCR4/Wnt/β-catenin pathway in tumor epithelial cells. CAFs secreting stromal growth factor- 1 (SDF-1 or CXCL12) have been shown to be the major players in this process. Moreover, SDF-1 is also interlinked with the resistance of tumor cells to chemotherapeutic agents such as cisplatin (Zhang et al., 2020).

In 2020, Franzè and colleagues demonstrated the activation of CAFs phenotype in normal fibroblasts from rectal polyps co-cultured with CAFs. They showed that CAFs derived from colorectal tumors can secrete IL-34, which in normal fibroblasts activates the expression of CAFs markers such as α-SMA, vimentin, and fibroblast activating protein (FAP) (Franzè et al., 2020).

Even though most of the described functions of СAFs are associated with the stimulation of tumor progression, some authors also describe СAFs as tumor-suppressing players. For example, the subpopulation of CAFs expressing the melanoma adhesion molecule (CD146) in breast tumors correlated with a retarded cell proliferation in estrogen-dependent types of breast cancer (Brechbuhl et al., 2017). Since CAFs secrete cytokines involved in the recruitment and maturation of macrophages, T-lymphocytes and natural killer cells (IL-10, TGF-β, TNF, IFN-γ and IL-6), they increase the availability of the tumor to immune cells and promote antitumor immune response (Marlow et al., 2008). In the study of oral cancer, it was also shown that CAFs can suppress the proliferation of tumor cells. In particular, the population of CAFs secreting Bone Morphogenetic Protein 4 (BMP4) and expressing α-SMA inhibited the proliferation of cancer stem cells (CSC) (Patel et al., 2018). Using the mice model with a predisposition for the development of pancreatic cancer, Rhim and colleagues exhibited the role of CAFs in cancer progression. They excluded the α-SMA-positive population of CAFs or CAFs with inhibited Hedgehog signaling pathway. These modifications suppress the growth of pancreatic ductal adenocarcinoma. Histological analysis of tumors revealed abnormalities in the vessel’s formation (Rhim et al., 2014).

These examples demonstrate the tumor-suppressing function of CAFs only in high differentiated cancers with no similarity in undifferentiated ones. It can be assumed that apart from origin and tissue of the affected organ, the function of CAFs is determined by the differentiation stage of cancer cells (Bu et al., 2019).

## Isolation of CAFs for research purposes

Cell cultures obtained from patients’ tumors after surgery are most often used to study the properties of CAFs. It is necessary to establish new CAFs cell cultures, since in vitro CAFs tend to age rapidly, and the possibilities of their use are limited by early passages (Taddei et al., 2014). Moreover, in commercially available cell collections American Type Culture Collection (ATCC), European Collection of Authenticated Cell Cultures (ECACC), Russian collection of cell cultures, etc. cell lines with the CAFs phenotype are limited. For instance, in ATCC, only one CAFs cell line is available. This cell culture originates from the prostate adenocarcinoma and is modified by the introduction of the telomerase transgene under the control of the constitutive promoter of the polyoma virus SV40 hTERT PF179T CAF. This modification of fibroblasts is aimed at maintaining the proliferative properties of CAFs (Madar et al., 2009).

To obtain cell cultures from tumor tissue, mechanical disaggregation, enzymatic dissociation, chelation and their combination are used. Trypsin and type IV collagenase are most often used to destroy the stroma of tumor tissue. The choice of what technique to use should consider the histological origin of the tissue of interest. When tissue disaggregated, CAFs can represent a small population of cells and obtaining a monoculture requires an additional stage of their separation from the total cell mass. In order to isolate a particular population of CAFs, magnetic separation, or FACS of cells with immunostaining of specific CAFs markers such as FAP or α-SMA are used (Sharon et al., 2013; Huang et al., 2017; Sha et al., 2018). The main difficulty in isolating CAFs lies in adapting protocols for vital staining of intracellular markers such as α-SMA, FAP, and vimentin. Therefore, it is highly desirable to include surface markers such as CD90 in the analysis.

## Conclusion

Clear understanding the tumor microenvironment role is crucial for the development of new approaches in cancer diagnostics and treatment. The multifaceted influence of CAFs on tumor progression makes them an important object for the study of carcinogenesis and the development of new antitumor agents. The use of drugs targeted to the components of the tumor microenvironment has not demonstrated efficacy for anti-metalloproteinase compounds and angiogenesis inhibitors as well as T-cell immunity checkpoints inhibitors in some types of cancer (Wang-Gillam, 2019). The heterogeneity of the molecular phenotypes of CAFs can be an important factor in the failure of CAF-targeted cancer treatment. The scientific community should develop more detailed classifications of various subtypes of CAFs considering their involvement in tumor progression.

Thus, a detailed classification of CAFs and a study of the functions of each phenotypic subgroup may provide important knowledge for the development of new methods for the CAF-related treatment and diagnosis of oncological diseases.

## Conflict of interest

The authors declare no conflict of interest.
